# Supplementary material for: Occurrence, risk factors and antimicrobial resistance of Campylobacter from poultry and humans in central Ethiopia: A one health approach
Source: PLoS Negl Trop Dis. 2025 Aug 11;19(8):e0012916. doi: 10.1371/journal.pntd.0012916 (PMC12360658; doi:10.1371/journal.pntd.0012916)
Supplement: S1 Table — A farm was considered Campylobacter-positive if the organism was isolated from at least one of the collected samples (human, poultry, or environmental) from that farm. (DOCX) [file pntd.0012916.s001.docx]

**Supplementary Table 1: Description of poultry farms in Debre Berhan and surrounding areas, and occurrence of *Campylobacter* at the farm level. A farm was considered *Campylobacter*-positive if the organism was isolated from at least one of the collected samples (human, poultry, or environmental) from that farm.**

| **Categories** | | | | **No. of poultry farm** | | ***Campylobacter* occurrence** | |
| --- | --- | --- | --- | --- | --- | --- | --- |
|  |  |  |  | **Frequency** | **Percent** | **Positive** | **Percent** |
| **Farm location** | Urban | | | 27 | 22.2 | 8 | 29.6 |
|  | Peri-urban | | | 78 | 63.9 | 29 | 37.2 |
|  | Rural | | | 17 | 13.9 | 2 | 11.7 |
| **Farming system** | Small holder | | | 58 | 47.5 | 15 | 25.8 |
|  | Commercial | | | 64 | 52.5 | 24 | 37.5 |
| **Farm type (by poultry kept)** | Broiler | | | 18 | 14.8 | 5 | 2.8 |
|  | Layer | | | 70 | 57.4 | 25 | 35.7 |
|  | Mixed | | | 6 | 4.9 | 3 | 50.0 |
|  | Grower | | | 28 | 22.9 | 6 | 21.4 |
| **Ventilation system** | Mechanical ventilation | | | 25 | 20.5 | 7 | 28.0 |
|  | Window | | | 79 | 64.8 | 25 | 31.6 |
|  | Under roof | | | 16 | 13.1 | 7 | 43.7 |
|  | No ventilation | | | 2 | 1.6 | 0 | 0.0 |
| **Biosecurity measures** | Rodent and insects control | | Yes | 51 | 41.8 | 19 | 37.3 |
|  |  |  | No | 71 | 58.2 | 20 | 28.2 |
|  | Use footbaths or change the shoes | | Yes | 33 | 27.1 | 7 | 21.2 |
|  |  |  | No | 89 | 72.9 | 32 | 35.9 |
|  | Restricted access to unauthorized personnel | | Yes | 70 | 57.4 | 19 | 27.1 |
|  |  |  | No | 52 | 42.6 | 20 | 38.5 |
|  | Disposal of poultry waste | | Backyard | 55 | 45.1 | 27 | 49.1 |
|  |  |  | Buried, burn or sell | 67 | 54.9 | 12 | 17.9 |
| **Separate sick poultry in the farm** | Yes | | | 66 | 54.1 | 19 | 28.8 |
|  | No | | | 56 | 45.9 | 20 | 35.7 |
| **Other animals kept in the farm** | No | | | 41 | 33.6 | 4 | 9.8 |
|  | Yes | Cattle | | 50 | 40.9 | 29 | 58.0 |
|  |  | Pet (dog and cat) | | 8 | 6.6 | 1 | 12.5 |
|  |  | Small ruminant | | 15 | 12.3 | 3 | 20.0 |
|  |  | Swine | | 8 | 6.6 | 2 | 25.0 |
| **Use of antibiotics as prophylactics** | Yes | | | 53 | 43.4 | 19 | 35.8 |
|  | No | | | 69 | 56.6 | 20 | 28.9 |
