# Supplementary material for: Occurrence, risk factors and antimicrobial resistance of Campylobacter from poultry and humans in central Ethiopia: A one health approach
Source: PLoS Negl Trop Dis. 2025 Aug 11;19(8):e0012916. doi: 10.1371/journal.pntd.0012916 (PMC12360658; doi:10.1371/journal.pntd.0012916)
Supplement: Attachment — Submitted filename: Response to reviewers.docx [file pntd.0012916.s003.docx]

**Point-by-Point Response to Reviewers’ Comments**

Reviewer #1: - The study was based in central Ethiopia. Figure 1 shows a clear map of the study area.
- It is clearly noted that the study is a cross-sectional approach of the population during March 2021 to March 2022 on selected poultry farms. The reasoning behind the study districts is mentioned due to the high concentration of these poultry farms. Overall, 122 poultry farms were used in the study to ensure statistical power and reliability. The author's have noted that professionals in the field were enlisted to help identify suitable poultry farms for investigation. The methods for choosing the poultry farms for the study are very clear, with calculations of sample size performed using a public software tool for epidemiology (CDC).
- Could the authors state how the chicken samples were randomly chosen (lines 143 and 175) and whether the number of samples was consistent across the different poultry houses. Was it similar to the lottery method used for the human samples? It would also be good for the authors to expand how this lottery method was performed for choosing the human samples when multiple people were present.

**Answer:** Due to the logistical challenge of assigning unique identifiers to individual chickens, we employed a random grab sampling method. We entered the poultry house and randomly caught a chicken without prior selection based on size, color, or location of each farm. For human participants, we implemented a lottery-based random selection method. Each available individual was assigned a unique number, and numbers were randomly drawn. The individuals whose numbers were selected were included in the study. This has been clarified in the manuscript. As we have indicated in the manuscript, the number of samples was consistent across all poultry farms. From each farm, we collected one poultry sample, one human sample, and one poultry house floor sample.

- Could the authors better link what they mean by 'sampled from all compartments of the poultry house floor' (lines 144-145) to the explanation on lines 178-183. I was able to gain better understanding of the approach after reading the later description. Was this kept consistent in terms of area and number of samples for each poultry house?

**Answer:** Thank you for your comment. We agree that the initial mention of "sampled from all compartments of the poultry house floor" (lines 144–145) could be clarified. To address this, we have revised the text to better link it to the detailed explanation provided in lines 178–183. Specifically, we clarify that one sock sample was collected per poultry house, and that sampling was performed according to Hansson et al. (2007), which involved walking the sock sample at least four times across the floor from wall to wall to ensure coverage of all compartments. This approach was applied consistently in all poultry houses included in the study.

- The author's clearly state that the structured questionnaire had open, closed, and semi-closed formatted questions (lines 157-158). The questionnaire was performed face-to-face using the local language, meaning that it was more likely that there would have been better response than a written questionnaire. The author's state that the questionnaire was pre-tested on ten farms to improve its clarity. Could the authors mention what ten farms these were and why/how they were chosen. How many improved questionnaires were then performed subsequently?

**Answer:** Thank you for your valuable comment. It has been included that the ten farms used for pre-testing the questionnaire were located in the same study area and were selected based on accessibility and willingness to participate. These farms were not part of the final study sample. Feedback from the pre-test was used to improve the clarity and flow of the questions. The final version of the questionnaire was then administered to 122 poultry farms included in the main study.

- Could the authors be specific/state whether there was a specific amount of hours for the person to provide a sample in (line 173).

**Answer**: It has been added that samples were provided within 2 hrs.

- Lines 180-181: is it possible for the authors to be more specific about walking through all areas of the farm floor multiple times. Were the number of times recorded and were they consistent for each farm? How did you ensure that they did not miss any areas?

**Answer:** Thank you for your comment. As described in the protocol by Hansson et al. (2007), which we followed, the sampler walked through all areas of the poultry house floor by walking the longest distance from wall to wall at least four times, ensuring comprehensive coverage. This procedure was applied consistently across all farms. While the number of passes was not individually recorded for each farm, the sampler was trained to follow the same standardized method at every site to minimize variation and ensure no areas were missed.

- Authors are overall very clear on how farm samples were stored before shipping and processing, as well as the isolation/identification of Campylobacter from the samples.
- Line 235: guideline would read better as plural (guidelines)
- Line 236: what do the authors mean by 'normal'? I think this word could be omitted, as I assume they just mean sterile saline.
- The authors describe well how the disc diffusion assays were performed, also noting that the discs were 20mm apart. This would have helped ensure that there was enough room for larger zones of clearing.
- Line 240: could the authors state specifically what the microaerobic conditions were ie the percentage of oxygen present.
- The authors mention the lack of EUCAST breakpoints for some antibiotics and that 0mm has been used as a cut-off for resistance. This is a conservative value, however, it is understandable why this was chosen in this instance. There are still no EUCASE breakpoints for the other antibiotics at the time of review.
- Methods overall clear to follow and well-written with only some minor adjustments required.
- Ethical considerations and approval, including the approval number, are clearly stated at the end of the methods.

**Answer**: Thank you for the minor comments included above. They have been corrected for.

Reviewer #2: Objectives were clearly stated and appropriate methodology used to address them, however there were some gaps in describing the study population and clarification needed in outlining the sampling locations. No concerns regarding ethical or regulatory requirements. The following outlines where more detail or clarification is needed:
• Clarification throughout that there were three study locations, rather than initially naming one then noting more further on in the text. Please specify which of the three locations are classified as rural/urban.

**Answer:** The study included urban (Debre Berhan town), peri-urban (outskirts of Debre Berhan town and Siyadebrina Wayu), and rural (Basona Worena) areas. Of the 32 poultry farms selected in Debre Berhan town, 27 were found in the urban and while 5, although within the Debre Berhan administrative boundaries, were situated on the outskirts and thus classified as peri-urban. In Basona Worena (rural area), 17 poultry farms were included in the study. In Siyadebrina Wayu, the peri-urban district surrounding Debre Berhan town, 73 poultry farms were included. This has been added in the manuscript.

• Line 140, unclear whether the inclusion criteria considered the size of the farm, urban/rural location or whether it was a commercial farm or small holding- please include if they were.

**Answer:** The inclusion criteria for poultry farms were being a smallholder or a commercial farm, along with accessibility and the farmers’ willingness to participate in the study. This has been added in the manuscript.

• Line 143, was there any effort to capture information from those who worked on the farm and lived elsewhere as well as those who lived and worked on the farm? If so at the moment this is not clear.

**Answer**: We did not distinguish between live-in and off-site workers, as their residence status was not the target of our investigation.

• Consistency in how the poultry house floor/ boot/sock samples are referred to throughout would help the reader follow the narrative better. (including in Fig 2).

**Answer**: Thank you for this comment. This has been corrected for.

• Line 155, was the epi data collected from the same individual who provided the human sample? Please clarify.

**Answer**: Yes, the epidemiological data was collected from the same individual who provided the human sample. This has been clarified in the revised manuscript.

• Line 162, clarification needed on epidemiological data collection i.e. did the same person interview all of the individuals included in the study and was a translator used?

**Answer**: All the questionnaires were administered by the same individual (the first author), who translated the questions orally into the local language (Amharic) during the interviews.

• Line 164, please specify whether questionnaire responses from the 10 farms the questionnaire was tested on were included in the analysis.

**Answer:** The responses from these 10 farms were not included in the final analysis, as they were part of the initial testing phase to refine the questionnaire.

• Line 180, as noted above, please ensure clarity on where the boot sock samples were taken- was it just in the poultry houses or throughout the farm as this line implies the latter and line 144 states ‘all compartments of the poultry house’

**Answer:** The manuscript has been corrected to ensure clarity. When we refer to ‘all compartments of the poultry house floor’ we specifically mean the floor area within the poultry house only. No samples were taken outside the poultry house floor.

• Line 191, please update heading, this section only refers to culture not identification.

**Answer**: Thank you. Corrected in the manuscript.

• Line 260, please add which direction was used for the step-wise model development, either forward or backward.

**Answer:** We used a forward-stepwise approach. This information has been added in the manuscript.

Reviewer #3: Authors of the study used the right methodology (sampling, microbiology analysis and statistic analysis)to acheived their objectives.

**Results**

Reviewer #1: - In the description of the poultry farms, it would be good to see a supplementary table for this. It would make it clearer to see which farms used antibiotics, had other animals, were in specific areas etc. This would also enable better links to be made between the conditions of the poultry farms and the Campylobacter isolated/identified.

**Answer:** Thank you for the suggestion. The farm-related data have been added as a supplementary table in the revised manuscript.

- Line 289: could the authors break down the percentage of oxytetracycline used on the farms out of the total 43.4% of antibiotics used.

***Answer:*** The commonly used antibiotics reported by farm owners included oxytetracycline, Tylosin Tartrate (ASHTYL 20% Powder), and Enrofloxacin (Ashinero 10% Oral Solution). All 53 farms mentioned oxytetracycline as a commonly used antibiotic. However, they did not consistently use a single type and indicated that usage depended on availability of the drug.

- Lines 297-299: very good noting of the Campylobacter spp. detected and break down of the sample percentages.

- Line 301: When it states 'on these farms' is it referring to the 39/122 poultry farms in the sentence before? Please make this a little more explicit.

**Answer:** This has been rephrased.

- Lines 304-306: you could add some percentages to these to supplement the raw numbers.

**Answer:** Thank you, this has been corrected for.

- Table 1 is a good addition to the text and supplements the results well
- Lines 316-321: Do the authors have any statistics for the individual remarks?

**Answer**: This has been added in the text.

- Line 322 states about the AOR/CI for an all-in/all-out approach, but it would be good to see how it breaks down individually, too, if possible. This way it will show us whether there is lack of one (or more) specific approach that contributes to higher Campylobacter prevalence.

**Answer**: We appreciate the reviewer's suggestion to examine the individual components of the 'all-in/all-out' approach. This management system is widely recommended by the World Organization for Animal Health (WOAH) as a comprehensive biosecurity practice, aimed at reducing disease transmission, including Campylobacter. It consists of synchronized introduction and removal of poultry of the same age cohort, followed by thorough cleaning and rest periods between batches. In this study, we analyzed the approach as an integrated package, rather than breaking down its individual elements, as these components are typically implemented together. While we do not have separate statistics for the individual elements, we recognize this as an important area for future research

- Table 3 should say Ratio not Ration (line 351)

**Answer:** Ok, Thank you.

- Table 3: Can the authors explain why there are only AORs given for 4 of the categories and not other categories?

**Answer**: At the bivariable logistic regression stage, the individual contribution of each independent variable to the outcome variable was assessed, and the results were expressed as crude odds ratios. Following this, multivariable logistic regression models were fitted for each of the explanatory variables with a p-value of less than 0.2 from the bivariate analysis. These models provide adjusted odds ratios for each explanatory variable, helping to identify the strongest predictors of the outcome. As a result, only four explanatory variables met the significance threshold and were retained in the final model, which is why AORs are presented only for these categories in Table 3. Variables that did not meet the significance criteria were excluded from the final analysis.

- How many respondents were there in total? The authors mention that 61.5% of respondents were unaware of zoonotic diseases (line 30). It would be good for them to add how many this was as a raw number as well.

**Answer**: This has been added.

- What is meant by ‘award respondents’? Line 361

**Answer**: Thank you for pointing this out. The term "award respondents" was intended to refer to those who were informed about zoonotic diseases. This has been corrected and clarified in the revised manuscript.

- Lines 385-386: a little hard to read at first. Please could the authors reword this for clarity. This could possibly be split into two separate sentences: 1) How many Campylobacter isolates were MDR and 2) How many were part of each species.

**Answer**: Thank you for the suggestion. We have revised the sentence for clarity by splitting it into two, as recommended. The revised version now clearly states the total number of MDR *Campylobacter* isolates, followed by the breakdown by species.

Lines 390-391 also slightly hard to understand. These sentences could also be broken up a little or re-worded. ‘All co-occurring C. jejuni strains on the same farms were MDR’ is fine as a stand-alone sentence. The 2nd sentence could be phrased such as ‘similar resistance patterns were observed across all sample types on five of six farms’. Having the original sentence of ‘except for isolates from one farm’ is a little confusing at the end.

**Answer:** Thanks, this has been corrected.

- Could the authors break table 4 down into resistance to all the singular antibiotics only as a separate table. This can then address the overall antibiotic resistance of the isolates, which can be referred to in-text, followed by MDR patterns of the combined antibiotics. Please can the authors add a 0 (or similar) to cells where there are no applicable isolates. The table currently looks unfinished without this.

**Answer**: This is a good idea, but the results from the singular antibiotics are already presented in the text under the heading ‘Antimicrobial resistance profiles’. It would be an overlap to present the results both in the text and in a new table. We had mark the cells with “-“ in table 4 with no applicable isolates.

Reviewer #2: Results presented are consistent with the approach outlined in the analysis plan but need reviewing to ensure accuracy of reported findings. On two occasions summarised in the text are not included as a table and p-values are not reported alongside COR/AORs. Tables and figures included are clearly laid out and look good.

**Answer:** Thank you for your feedback. We reviewed the results carefully to ensure the accuracy of the reported findings and ensured that p-values were reported alongside the COR/AORs as required.

The following outlines where more detail or clarification is needed:
• Lines 280 to 284, please ensure percentages quoted add up to 100%, in these lines they only total 99.9% each time.

**Answer:** The percentages have been corrected to total 100% in the revised manuscript.
• Data presented in lines 280 to 290 should be included as a table.

**Answer:** The data presented have been formatted as a supplementary table in the revised manuscript.

• Line 299, please check calculation of percentage of isolates which were C. coli, 9/46=19.6% not 24.3%.
• Line 316 to 319, reference table 2 and include CORs in the text so evidence of the significant results are clear, would also be beneficial to indicate where the strongest associations are- e.g. cattle/all-in-all-out management.

• Lines 322 and 325, please include p-value with the AOR.

• Lines 338 to 342, please include CORs in the text so evidence of the significant results are clear.

• Lines 344 to 348, please include p-value with the AOR.

**Answer**: Thanks for the minor comments above. They have all been corrected for.

• Lines 360 to 370, data presented in the text should be summarized in a table too, might be beneficial to include as a supplementary.

**Answer**: Thank you for the suggestion. The farm-related data have been added as a supplementary table in the revised manuscript.

• Line 391, please explain the resistance pattern from isolates on the one farm which had different AMR

**Answer:** This data has been added in the revised manuscript.

Reviewer #3: They used the right statistical analysis to identify to risk factors associated to antimicrobial resistance of Campylobacter from Poultry and Humans in Central Ethiopia. Results have been clearly presented, tables are sufficient quality, but the quality of image of the map need to improve.

**Conclusions**

Reviewer #1: - Campylobacter jejuni is noted as critical in human infection and links to Campylobacter in other studies, including Ethiopia, has been noted and discrepancies addressed. There were also links made between higher Campylobacter incidence in farms where there is both poultry and cattle, which corroborates that of previously published articles. The authors have noted that this could be due to horizontal transmission. They highlight domestic animals as a source of Campylobacter transmission to humans.
- The authors clearly link a lot of Campylobacter risk factors identified in the study to other global studies. For example, drinking raw milk, poor hygiene/lack of PPE, and allowing poultry to freely roam in food preparation areas. They have highlighted the requirement for education of farm workers around zoonotic diseases and the One Health concept. They note in the conclusions about future interventions within Ethiopia which may contribute to reducing Campylobacter risks.
- The discussion mentions about antimicrobial resistance being a major concern in the study, with all Campylobacter being resistant to at least one antibiotic. The authors have done well to link the findings from other studies both within Ethiopia and globally. The authors could also mention the limitations of the methods/results due to the lack of EUCAST break points for some antibiotics. Alternative methods such as minimum inhibitory concentrations and E-test strips could be mentioned but noted that these are either not time-effective or can be costly, and thus the disc diffusion assay was still the best choice for this study.
- I would like to see the authors address the limitations of their study a little clearer within the discussion. Whilst they make some very valid points and links to previous literature, there are potentially some limitations within the methods and results around the sampling of the poultry and the houses.

Reviewer #2: Conclusions drawn from the findings presented are supported and authors have clearly demonstrated how this study adds to the current evidence, which is somewhat limited. Novel One Health approach taken is very relevant and provides a good framework for future studies to investigate this topic in more detail. Findings are clearly linked to potential public health actions which can be taken to reduce human/animal health risks.

Reviewer #3: The conclusion support presented data. No limitation of analysis has been mentioned. The public health relevance of data has been addressed.

**Response to Reviewers #1 and #3:**
Thank you for your comments. The limitations of the study have been clearly addressed in the revised manuscript, as suggested. Please see the updated "Limitations of the Study" section.

**Editorial and Data Presentation Modifications?**

Reviewer #1: Minor revisions with some expansions clarifying the comments above

Reviewer #2: This was a well written manuscript with relevant references included and only a few typos. I have outlined below where either further clarification or expansion on a point to provide more context would be better:

• Typos on line 103 (also), in Figure 1 (‘town’ in bottom panel), 361 (aware)
• Line 76, these references are for northern European countries only, please make this clear in the text or include references from a more directly comparable location• Line 79- text here is a direct quote, but not indicated- please make this clear
• Line 92, more information on the context of the AMR rates for Campylobacter in Ethiopia would be helpful- were the samples taken from humans/poultry/livestock, when and was there a trend over time? Give the range quoted is 0% to 100% more information is needed to understand how applicable to the study and also may be more important to stress that estimates are variable/little is known
• Line 104, would suggest that this sentence is aligned with the abstract to also include the environment.
• Clarification on ‘all-in-all-out management’ meaning

**Answer**: All the comments above have been corrected for.

• Line 391, It would be very interesting to know whether there was any pattern in resistance by type of farm (commercial vs small holding) or location (e.g. urban vs rural).

**Answer**: We appreciate the reviewer’s suggestion. While investigating resistance patterns by farm type or location would indeed be insightful, our current dataset did not allow for reliable subgroup analysis. Dividing the data into smaller categories would have resulted in very small sample sizes per group, limiting the statistical validity of any observed patterns. We agree this is an important area for further investigation and recommend that future studies with larger and more balanced samples explore these potentially meaningful differences.

• Line 469 to 470- you note nearly half the farms in the study area’ reply on antibiotic use a prophylaxis, did you collect information on antibiotic use for each farm recruited to the study, if not this should be noted as a limitation

**Answer**: Yes, we collected information from each farm regarding whether they use antibiotics as prophylaxis. These data are included in the Manuscript.

Reviewer #3: (No Response)

**Summary and General Comments**


Use this section to provide overall comments, discuss strengths/weaknesses of the study, novelty, significance, general execution and scholarship. You may also include additional comments for the author, including concerns about dual publication, research ethics, or publication ethics. If requesting major revision, please articulate the new experiments that are needed.

Reviewer #1: I feel that this is a strong submission of Campylobacter epidemiology in Ethiopia. It is of particular interest as Campylobacter is under sampled in this area, so this paper will contribute well to the research field and the PLOS Neglected Tropical Diseases journal.

If the authors can address my minor comments and recommendations above, I believe this will be worthy of publication.

Reviewer #2: I really enjoyed reading this manuscript and I think it's a brilliant study which will not only add to the international evidence base but also provides a design which could easily be replicated in other countries to offer further insights. This work further highlights the importance of a One Health approach and was a well designed study. However, the manuscript would benefit significantly from a few revisions to ensure accuracy, clarity and also that all data presented in the text are available to the reader alongside for their consideration.

Reviewer #3: Campylobacter is a major cause au diarrheal diseases worldwide, and results you presented have a significance importance in public health.

I appreciated the effort you made to discussion yours results.


What version of EUCAST did you used to interpret the antimicrobial suseptibility test regarding oxytetracycline, streptomycin, nalidixic acid?

Can you pleased summarized title of tables you used in the results part? They are too longs.

You can now used legend to explain what you want.

Tiltle of figure 1 can be improve and be precise.

**Answer**: Thanks for the suggestions above, they have been corrected for.

The authors wish to thank all three reviewers for good comments, which have improved the MS.
