# Supplementary material for: Occurrence, risk factors and antimicrobial resistance of Campylobacter from poultry and humans in central Ethiopia: A one health approach
Source: PLoS Negl Trop Dis. 2025 Aug 11;19(8):e0012916. doi: 10.1371/journal.pntd.0012916 (PMC12360658; doi:10.1371/journal.pntd.0012916)
Supplement: Attachment — Submitted filename: Response to Reviewer.docx [file pntd.0012916.s004.docx]

Response to Reviewer

Dear Reviewer,

We thank you for the opportunity to revise and resubmit our manuscript entitled **“Occurrence, Risk Factors and Antimicrobial Resistance of *Campylobacter* from Poultry and Humans in Central Ethiopia: A One Health Approach”** (Manuscript ID: **PNTD-D-25-00226R1**) to *PLOS Neglected tropical diseases*. We are grateful for the constructive feedback provided by the editorial team and reviewers, which has greatly helped us improve the manuscript.

All required figures were processed through the PACE tool to ensure compliance with PLOS formatting standards. The updated PACE-validated files have been uploaded, replacing previous versions. Supporting Information files were excluded, as per the guidelines. We thank the PLOS reviewer for recommending the use of the PACE tool to help us meet the journal’s quality standards.

We appreciate the recommendation to enhance the reproducibility of our results. In this study, the organism was isolated following ISO 10272, confirmed using multiplex PCR, and tested for antimicrobial susceptibility using the disc diffusion method according to EUCAST guidelines. As these methods are standardized and widely accessible, we believe they provide a reproducible framework for similar studies. However, we support open science practices and will consider depositing any relevant protocols in protocols.io for future work where novel or non-standard procedures are applied.

We believe that all editorial and reviewer comments have now been addressed. We respectfully submit the revised manuscript and look forward to your decision.

Thank you again for your support and consideration.

Sincerely,
**Fikre Zeru**
*On behalf of all co-authors*
